# Aberrant glioblastoma neovascularization patterns and their correlation with DCE-MRI-derived parameters following temozolomide and bevacizumab treatment

**DOI:** 10.1038/s41598-017-14341-9

**Published:** 2017-10-24

**Authors:** Wei Xue, Xuesong Du, Hao Wu, Heng Liu, Tian Xie, Haipeng Tong, Xiao Chen, Yu Guo, Weiguo Zhang

**Affiliations:** 1 Department of Radiology, Institute of Surgery Research, Daping Hospital, Third Military Medical University, Chongqing, 400042 China; 2 Chongqing Clinical Research Center for Imaging and Nuclear Medicine, Chongqing, 400042 China

## Abstract

Glioblastoma (GBM) is a highly angiogenic malignancy, and its abundant, aberrant neovascularization is closely related to the proliferation and invasion of tumor cells. However, anti-angiogenesis combined with standard radio-/chemo-therapy produces little improvement in treatment outcomes. Determining the reason for treatment failure is pivotal for GBM treatment. Here, histopathological analysis and dynamic contrast-enhanced MRI (DCE-MRI) were used to explore the effects of temozolomide (TMZ) and bevacizumab (BEV) on GBM neovascularization patterns in an orthotopic U87MG mouse model at 1, 3 and 6 days after treatment. We found that the amount of vascular mimicry (VM) significantly increased 6 days after BEV treatment. TMZ inhibited neovascularization at an early stage, but the microvessel density (MVD) and transfer coefficient (K^trans^) derived from DCE-MRI increased 6 days after treatment. TMZ and BEV combination therapy slightly prolonged the inhibitory effect on tumor microvessels. Sprouting angiogenesis was positively correlated with K^trans^ in all treatment groups. The increase in VM after BEV administration and the increase in MVD and Ktrans after TMZ administration may be responsible for treatment resistance. K^trans^ holds great potential as an imaging biomarker for indicating the variation in sprouting angiogenesis during drug treatment for GBM.

## Introduction

Glioblastoma (GBM) is the most common primary malignant brain tumor in adults. Despite advances in tumor therapy, such as maximum surgical resection followed by radio-/chemo-therapy and immune/gene therapy^1^, the prognosis of GBM patients is still poor, consisting of a median survival time of 14.6 months and a five-year survival rate lower than 10%^2,3^. GBM is characterized by abundant and abnormal neovascularization, including vascular co-option, angiogenesis, vasculogenesis, mosaic vessel formation, vascular mimicry, and glioblastoma-endothelial cell transdifferentiation^4–7^. Additionally, the neoplastic microvessels are leaky, endothelial cells are aberrant, and the pericytes and basement membrane are absent^8^ leading to a hypoxic and acidic microenvironment, which is closely related to tumor progression, metastasis and relapse^9,10^.

As a humanized monoclonal antibody against vascular endothelial growth factor (VEGF), bevacizumab (BEV) can block VEGF signal transduction to generate an anti-tumor effect by inhibiting neovascularization and suppressing edema^11,12^, but as a result, BEV can also increase tumor cell invasion in GBM^13^. Temozolomide (TMZ) is an alkylating agent that promotes apoptosis and is used as a first-line chemotherapeutic agent against newly diagnosed GBM^14^. The use of TMZ could improve treatment for GBM^2^. However, resistance to TMZ impairs its therapeutic effect, and the mechanisms of TMZ resistance are still not clearly understood. Meanwhile, TMZ has been reported to inhibit tumor angiogenesis^15^. However, it is not clear which types of neovascularization patterns are sensitive to TMZ and BEV treatment.

Pre-clinical studies are an important approach to explore tumor neovascularization patterns. For example, vascular co-option has been demonstrated in a rat C6 glioma model^16^. Vasculogenesis has been further demonstrated in the angiogenic, defective tumor resistant Id-mutant mouse model^17^. Glioblastoma-endothelial cell transdifferentiation has been shown in orthotopic GBM mice models^18^. Using orthotopic rat C6 glioma models, our group discovered a new mosaic pattern of glioma vascularization^5^ and confirmed vascular co-option, sprouting angiogenesis, intussusceptive microvascular growth (IMG) and vascular mimicry in the tumor region. We also demonstrated that neovascularization patterns varied along with tumor development and that dynamic contrast-enhanced MRI (DCE-MRI) can be used to evaluate neovascularization patterns in a glioma model^19^.

Vascular parameters can be dynamically evaluated by MRI *in vivo*. Dynamic susceptibility contrast MRI (DSC-MRI) can assess tumor vessel perfusion, and DCE-MRI can be used to evaluate vascular permeability^20^. DCE-MRI measures the MR signal to determine the change in concentration of the contrast agent over time within a field of view (FOV), and its quantitative parameters can be obtained through different pharmacokinetic models. For example, K^trans^ reflects vessel permeability, V_p_ reflects plasma volume, and K_ep_ reflects regurgitation of contrast media^21^. K^trans^ has been reported to be strongly correlated with glioma vascular permeability and microvessel density (MVD) after anti-angiogenesis therapy^22,23^. Therefore, we used DCE-MRI to monitor the effect of anti-angiogenesis therapy in this study.

Recently, many studies have demonstrated that different neovascularization patterns are mediated by corresponding signaling pathways^24,25^ with varying sensitivities to anti-angiogenic drugs^26,27^. However, few studies have examined the change in neovascularization patterns after treatment for GBM. In our study, orthotopic U87MG glioblastoma mouse models were administered TMZ, BEV or a combination of BEV and TMZ, and then DCE-MRI scanning and histopathological analysis were performed to investigate the changes in neovascularization patterns and MVD in the tumor region. We aimed to determine the relationship between neovascularization patterns and resistance to drug treatment and investigated MRI biomarkers that indicate changes in neovascularization patterns during treatment.

## Results

### MRI features and neovascularization patterns of a U87 GBM model

**Figure 1:**
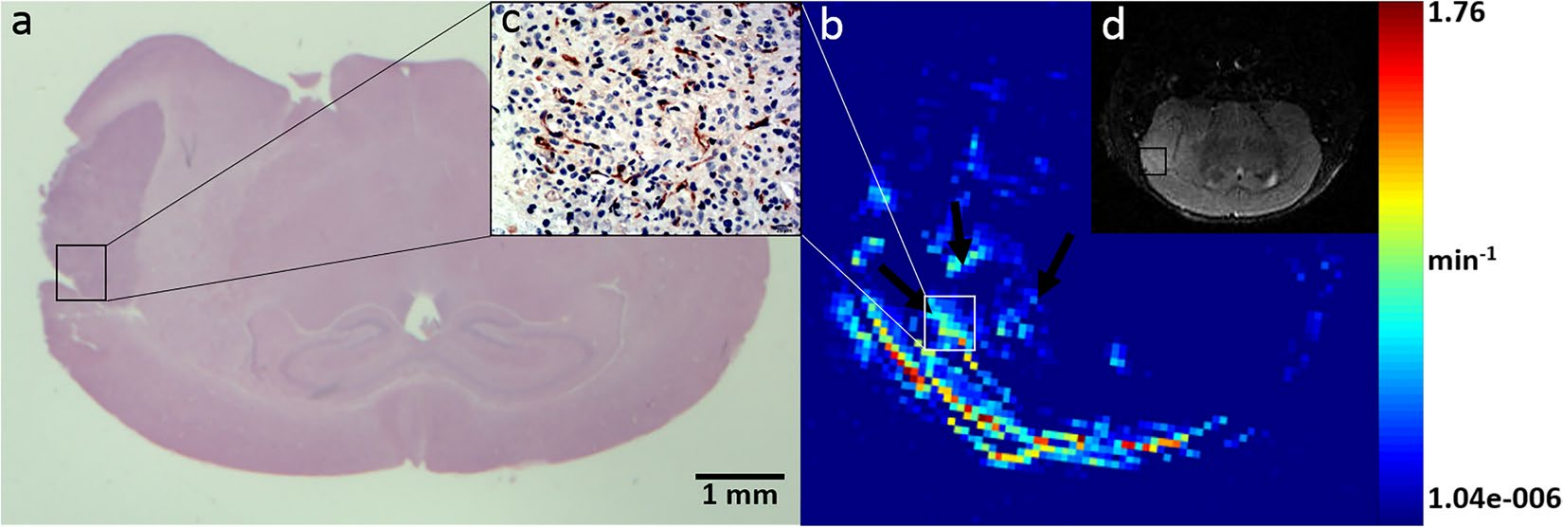
MRI and corresponding histopathological features of the orthotopic U87MG glioblastoma model. HE staining (**a**) and a T2-weighted image (**d**) show a quasi-circular mass in the right cerebral hemisphere with mild edema around the tumor mass. The K^trans^ map (**b**) of the tumor is shown. Bright points in the tumor area represent high permeability, especially in the tumor margin, which also showed more abundant neovascularization (**c**). All images were selected from the maximum cross-section of the tumor.

GBM presented as a quasi-circular high signal in the right cerebral hemisphere in T2-weighted images. The signal was homogeneous, and mild edema was observed around the tumor mass. The K^trans^ map derived from DCE-MRI showed high permeability in tumor tissues, especially at the tumor margin, indicated by bright points (Fig. 1). Four types of neovascularization patterns were identified by pathological staining of the tumor region, including vascular co-option, sprouting angiogenesis, IMG and vascular mimicry.

### Histopathological results

**Figure 2:**
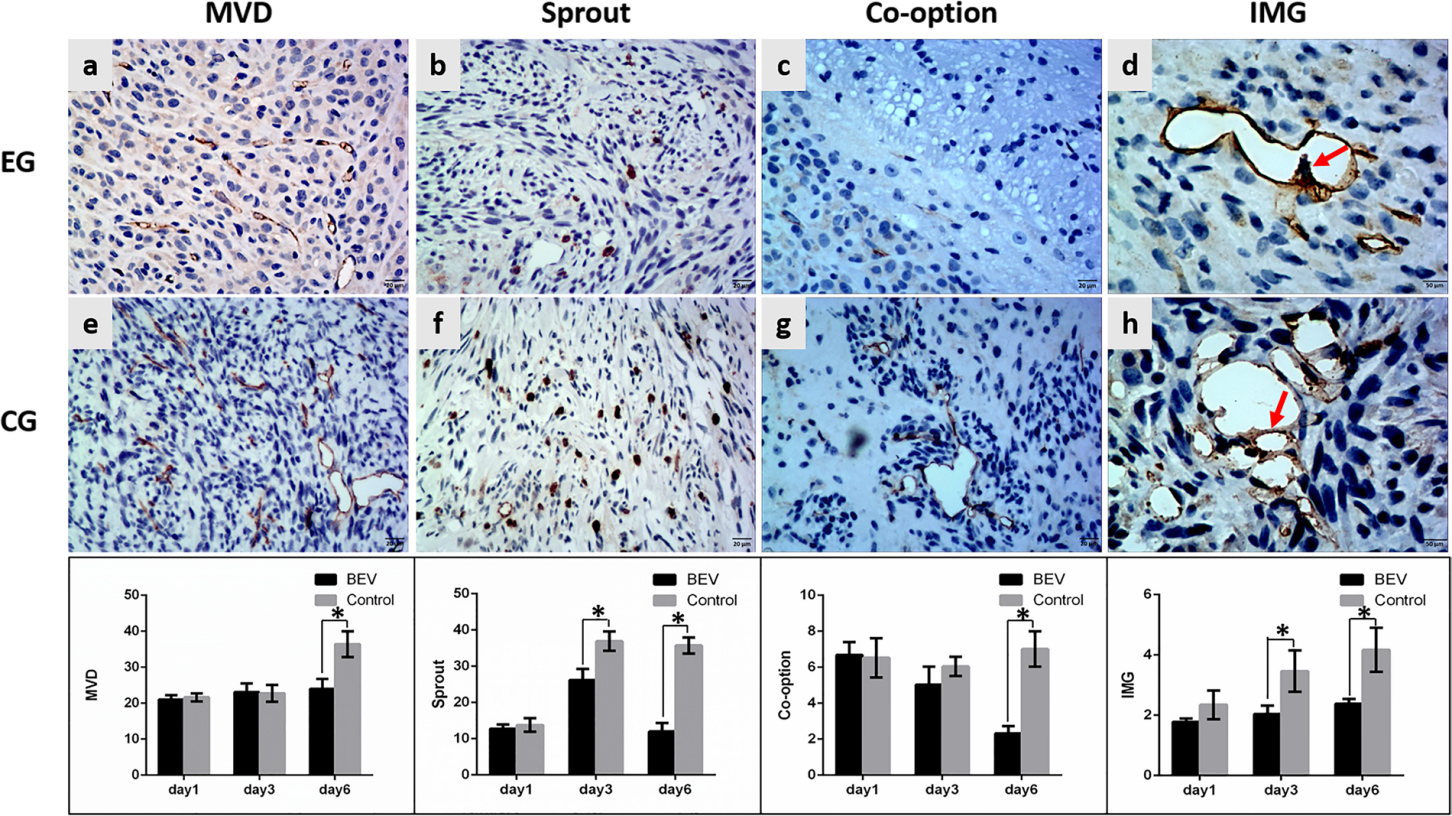
The variation in neovascularization patterns after BEV administration. The histopathological images show the variation in MVD, sprouting angiogenesis, co-option and IMG at 6 days after BEV administration. Upper row, experimental group (EG), middle row, corresponding control group (CG). The lower bar graphs show the variation in corresponding neovascularization patterns after BEV administration at three time points. (**a**,**e**) CD34-positive tumor microvessel. (**b**,**f**) Tenascin-C-positive stalk representing vascular sprouting. (**c**,**g**) Tumor cells surrounding CD34-positive tubes represent vascular co-option. (**d**,**h**) CD34-positive folds across the lumens represent intussusceptive microvascular growth (red arrow). Data were represented as the means ± SD. *Indicates P < 0.05.

**Figure 3:**
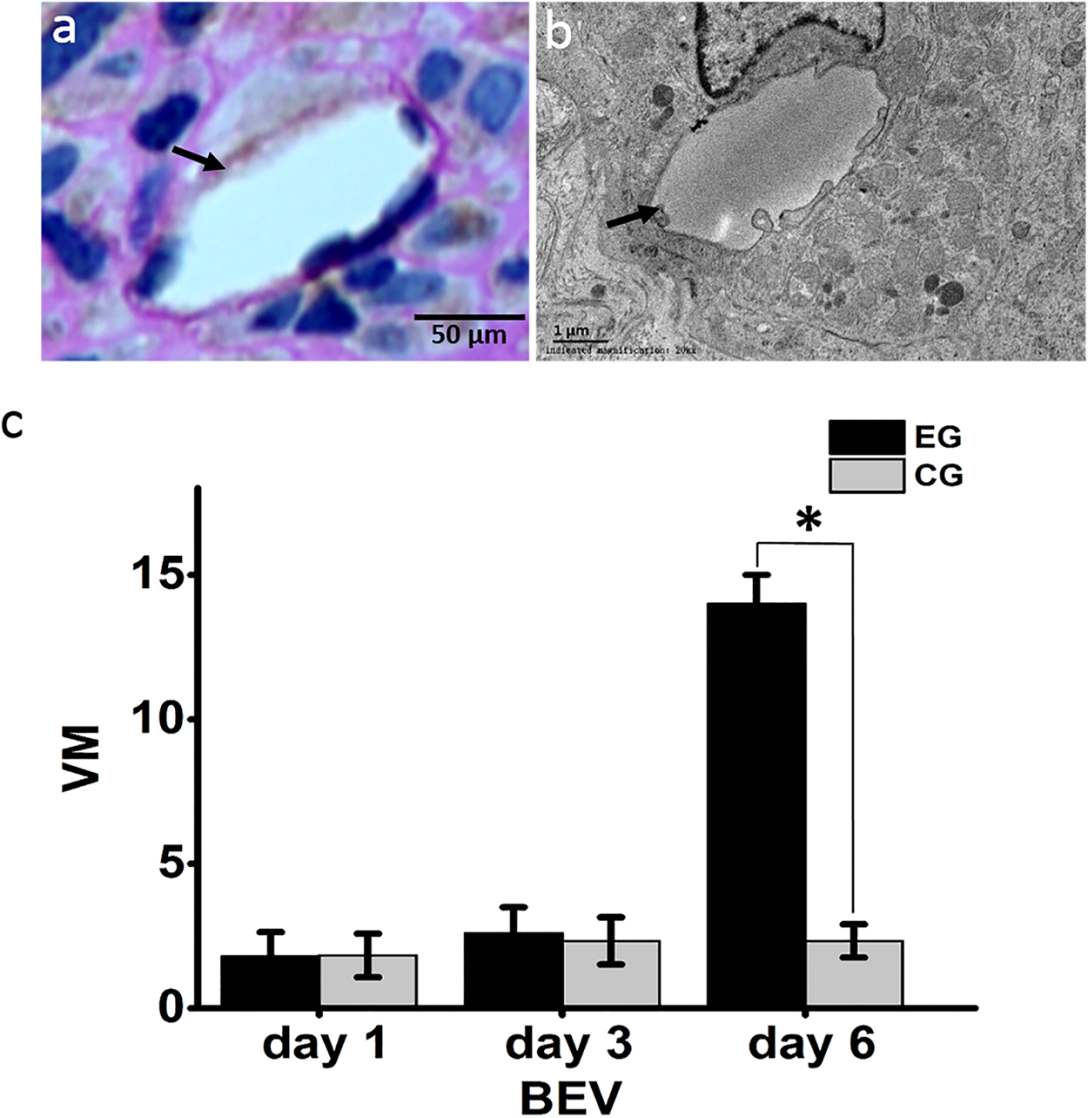
Vascular mimicry. (**a**) Vascular mimicry was characterized by PAS-positive and CD34-negative lumen-like structures (black arrow). (**b**) Transmission electron microscopy shows tumor cells lining a vascular channel (black arrow). (**c**) The amount of vascular mimicry (VM) was significantly increased 6 days after BEV administration, experimental group (EG), control group (CG). Data were represented as the means ± SD. *Indicates P < 0.05.

**Figure 4:**
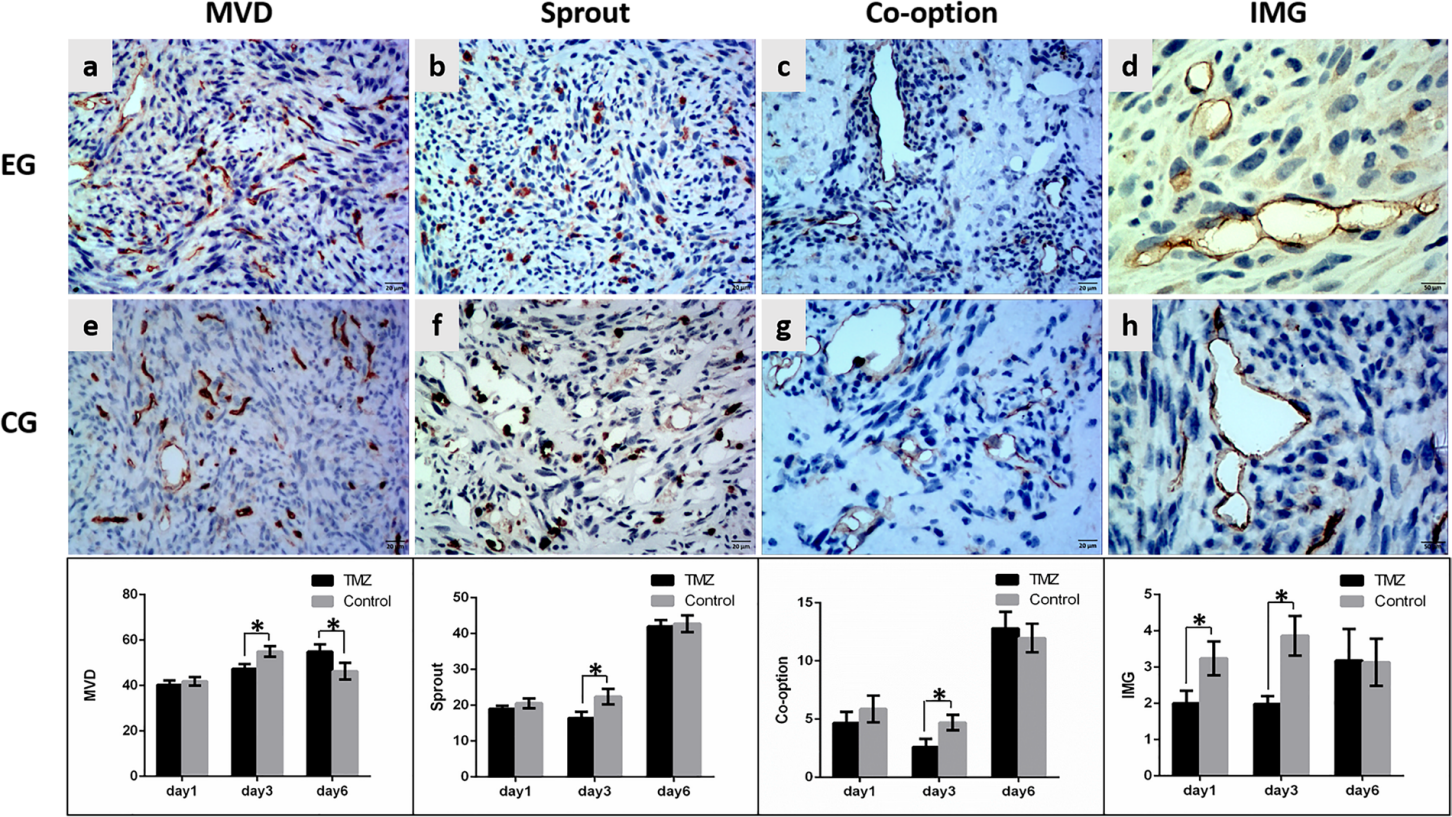
The variation in neovascularization patterns after TMZ administration. The histopathological images show the variation in MVD, sprouting angiogenesis, co-option and IMG at 6 days after TMZ administration. Upper row, experimental group (EG), middle row, corresponding control group (CG). The lower bar graphs show the variation in corresponding neovascularization patterns after TMZ administration at three time points. Data were represented as the means ± SD. *Indicates P < 0.05.

**Figure 5:**
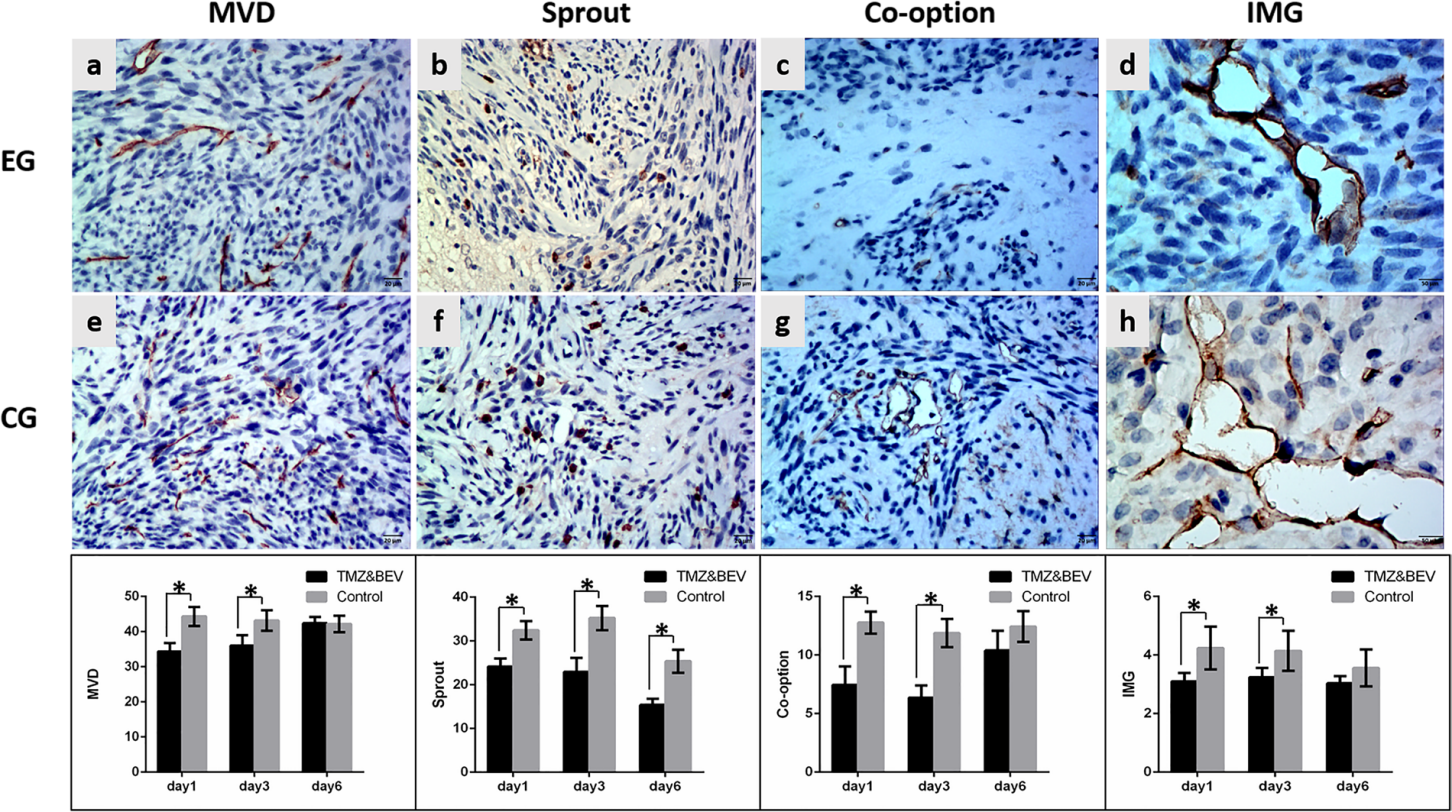
The variation in neovascularization patterns after a combination of BEV and TMZ administration. The histopathological images show the variation in MVD, sprouting angiogenesis, co-option and IMG at 3 days after BEV and TMZ administration. Upper row, experimental group (EG), middle row, corresponding control group (CG). The lower bar graphs show the variation in corresponding neovascularization patterns after the combination administration at three time points. Data were represented as the means ± SD. *Indicates P < 0.05.

The reproducibility of the histopathological results were assessed using the intraclass correlation coefficient (ICC = 0.95). Different treatment protocols had varying effects on tumor neovascularization patterns. In the BEV group, BEV had no effect on the MVD or neovascularization patterns 1 day after treatment. The amount of sprouting angiogenesis and IMG began to decrease 3 days after treatment compared with control groups (P < 0.001, P = 0.002, respectively). The MVD and the amount of IMG were both decreased compared with control group (P < 0.001, P = 0.001, respectively) and an actual decrease was observed in the number of co-option and sprouting angiogenesis 6 days after treatment (P < 0.001, P < 0.001, respectively) (Fig. 2), whereas the amount of vascular mimicry was significantly increased (P < 0.001) (Fig. 3). In the TMZ group, TMZ inhibited tumor neovascularization 3 days after treatment. The amounts of sprouting angiogenesis, IMG and MVD were all significantly decreased compared with control groups (P = 0.001, P < 0.001, P < 0.001, respectively) and an actual decrease was observed in the number of vascular co-option (P = 0.001) 3 days after treatment. IMG was the most sensitive neovascularization pattern to TMZ and decreased 1 day after treatment compared with control groups (P = 0.001). However, 6 days after treatment, the MVD was significantly increased compared to the control group (P = 0.003), and no significant differences were found in the amounts of vascular co-option, sprouting angiogenesis, IMG and vascular mimicry between the experimental group and the control group (Fig. 4). In the BEV and TMZ combined group, the combination of BEV and TMZ significantly reduced the MVD and the amount of vascular co-option, sprouting angiogenesis and IMG 1 day (P < 0.001, P < 0.001, P < 0.001, P = 0.010, respectively) and 3 days (P = 0.003, P < 0.001, P < 0.001, P = 0.025, respectively) after treatment compared with control groups, but the amount of vascular mimicry (VM) was not different between the experimental and control groups. However, 6 days after treatment, the only decrease was observed in sprouting angiogenesis (P < 0.001) (Fig. 5).

### DCE-MRI and correlation analysis

**Figure 6:**
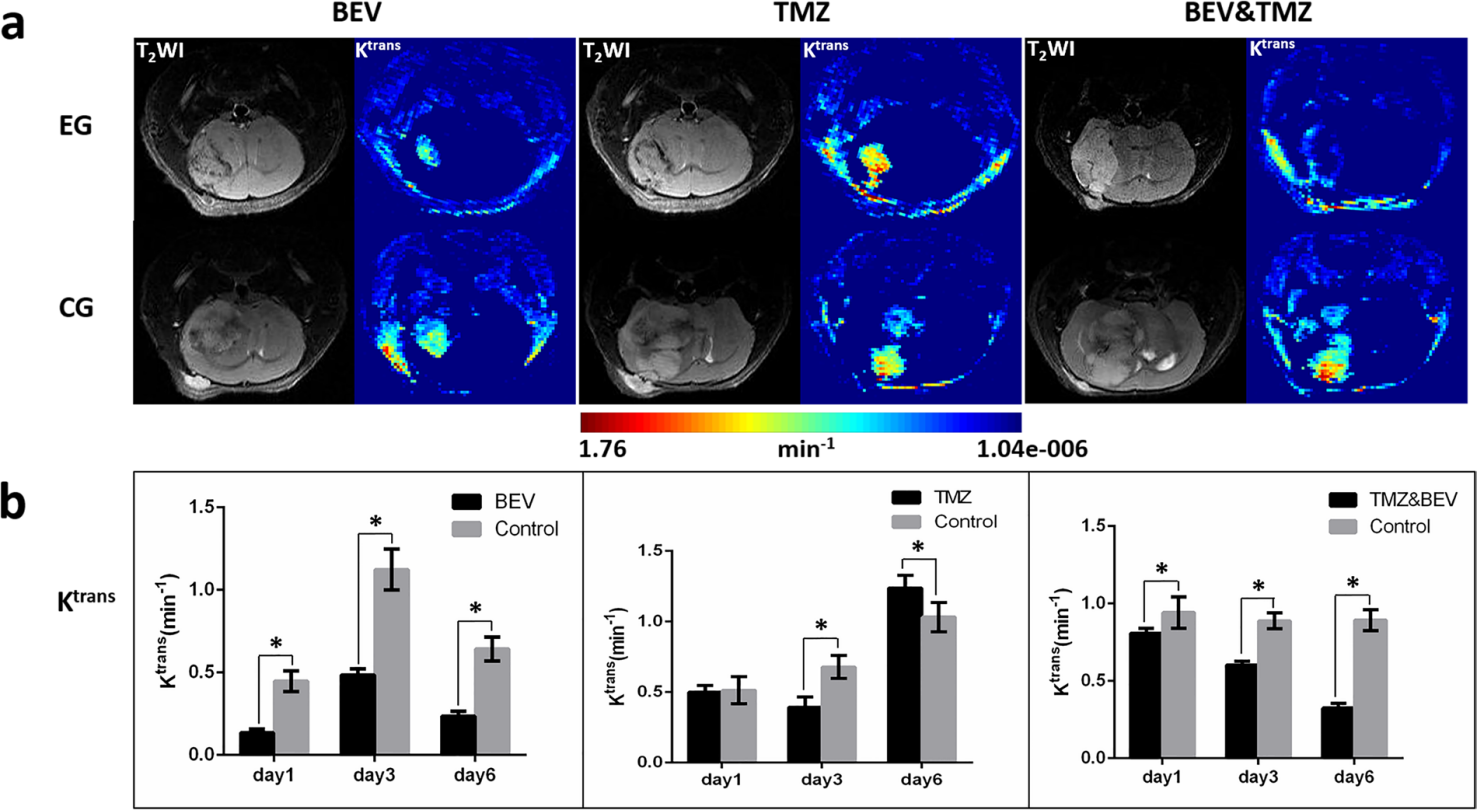
The variation in K^trans^ after treatment. (**a**) T_2_-weighted images (T_2_WI, left column) and corresponding K^trans^ maps (right column) of GBM-bearing mice at 6 days after various treatments. Upper row, experimental group (EG); lower row, control group (CG). (**b**) K^trans^ values at different time points in three treatment groups. Data were represented as the means ± SD. *Indicates P < 0.05.

**Figure 7:**
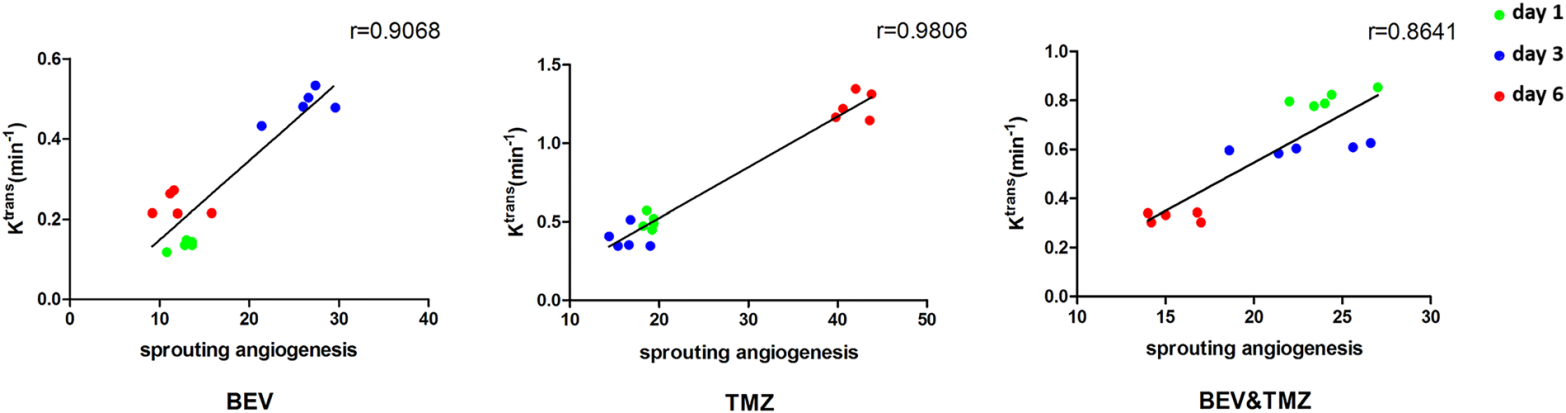
The amount of sprouting angiogenesis was positively correlated with K^trans^ value in experimental groups after three kinds of treatments.

The reproducibility of the DCE-MRI results were assessed using the intraclass correlation coefficient (ICC = 0.94). In the BEV treatment group, the K^trans^ significantly decreased from day 1 through day 6 after treatment compared with control groups (P < 0.001 at 1, 3 and 6 days, respectively). In the TMZ treatment group, K^trans^ significantly decreased 3 days after treatment but increased on the 6th day after treatment compared with control groups (P < 0.001, P = 0.007, respectively). In the BEV and TMZ combination treatment group, the variation in K^trans^ was similar to that in the BEV treatment group. K^trans^ was significantly reduced from day 1 to day 6 after treatment compared to the control groups (P = 0.022, P < 0.001, P < 0.001 at 1, 3 and 6 days, respectively) (Fig. 6). Spearman correlation analysis showed that K^trans^ was positively correlated with sprouting angiogenesis in all experimental groups after drugs administration and had no correlation with MVD, co-option, IMG, or VM (All the P values < 0.001) (Fig. 7).

## Discussion

GBM is a highly angiogenic malignancy, and one of the characteristics of GBM is abundant and aberrant neovascularization. These new blood vessels provide nutrition and oxygen for tumor growth and are also closely related to the proliferation and invasion of tumor cells^7^. However, the failure of anti-angiogenesis therapy and the resistance to TMZ treatment in patients with GBM highlight clinical dilemma that motivated us to explore a novel perspective for the treatment of GBM. In this study, we found that TMZ could inhibit neovascularization at an early stage but subsequently led to an increase in MVD and K^trans^. BEV could increase the amount of VM 6 days after treatment, which could be responsible for the failure of anti-angiogenesis and standard TMZ treatment in patients with GBM. In addition, we found that sprouting angiogenesis was positively correlated with K^trans^ in all treatment groups, indicating that K^trans^ holds great potential as an imaging biomarker to indicate the variation in sprouting angiogenesis during drug treatment for GBM.

We found that TMZ suppressed the neovascularization in orthotopic U87MG GBM mouse models, which was consistent with the research reported by Hjalmar Kurzen^28^. The MVD and the amount of vascular co-option, sprouting angiogenesis and IMG were obviously decreased on the 3rd day after treatment in the tumor region. However, on the 6th day after TMZ administration, the amounts of vascular co-option, sprouting angiogenesis, IMG and vascular mimicry were not different between the experimental and control groups. The MVD and K^trans^ even increased, suggesting that exuberant angiogenesis in GBM might be a crucial cause of chemo-therapy resistance and also demonstrating the necessity of anti-angiogenesis in the treatment of GBM.

BEV can inhibit neovascularization by competitively binding to VEGF and blocking its biological activity. Previous studies have shown that anti-angiogenesis treatment increases tumor cell invasion and vascular co-option^29–31^. In the present study, BEV began to reduce the amount of sprouting angiogenesis and IMG 3 days after treatment. The MVD and the amount of co-option, sprouting angiogenesis and IMG were all decreased on the 6th day after treatment, whereas the amount of vascular mimicry was significantly increased, indicating that vascular mimicry may precede tumor cell invasion and vascular co-option in response to anti-VEGF therapy. Vascular mimicry is associated with the prognosis of laryngeal cancer^32^, breast cancer^33^ and glioblastoma^34^, suggesting that the increase in vascular mimicry is an important reason for the failure of BEV treatment at an early stage.

By dynamically monitoring the distribution of contrast media in the region of the tumor blood vessels and the tissue space, DCE-MRI can directly or indirectly reflect the structure and function of tumor blood vessels^21^. DCE-MRI has been widely used for early diagnosis^35^ and evaluating therapeutic efficacy^36^. K^trans^, a major parameter of DCE-MRI, reflects vascular permeability and has been used as one of the main indicators for the clinical evaluation of anti-tumor drugs^37^. In our study, we utilized DCE-MRI to evaluate the effect of TMZ and BEV on GBM neovascularization patterns. Numerous studies have demonstrated that the breakdown of normal vascular structures by tumor cells is not only an important process to increase blood supply but also an important indicator of tumor invasion and metastasis^7^. Sprouting angiogenesis is a major modality for tumors to generate new vessels from preexisting ones. We found that sprouting angiogenesis was positively correlated with K^trans^ in all treatment groups, suggesting that K^trans^ is a promising imaging biomarker for indicating changes in sprouting angiogenesis after treatments.

In this study, we found that K^trans^ significantly decreased from day 1 to day 6 after BEV treatment compared with control groups. According to previous reports, normalization of vascular abnormalities emerged after BEV administration and could improve the effect of radio-/chemo-therapy^38^. Our results are consistent with the hypothesis that K^trans^ is an imaging biomarker of vascular normalization.

The addition of BEV to standard radio-/chemo-therapy after surgery has been reported to relieve clinical symptoms and prolong progression-free survival but not overall survival in patients with GBM^39^. In our observations, the greatest anti-angiogenesis effect in orthotopic U87MG GBM mouse models was achieved 3 days after combined TMZ and BEV administration (TMZ treatment was administered 24 hours after the administration of BEV), suggesting that an optimization treatment cycle could be determined for clinical management to achieve the greatest inhibitory effect on tumor neovascularization and growth.

Nevertheless, our study has some limitations. Patient-specific orthotopic GBM xenograft models should be used to accurately reflect the histological and genetic characteristics of the primary tumor and mimic the treatment response of parental GBM^40^.

In conclusion, we found that TMZ could stimulate neovascularization 6 days after treatment, and the amount of VM was increased 6 days after BEV treatment, which may be responsible for treatment failure. K^trans^ can non-invasively indicate the variation in sprouting angiogenesis and reflect the function of tumor microvessels during anti-angiogenic treatment.

## Methods

### U87GM cell culture

The human glioma U87GM cell line was purchased from the Type Culture Collection of the Chinese Academy of Sciences (Shanghai, China). Cells were cultured at 37 °C in 5% carbon dioxide in high-glucose DMEM (Gibco, Carlsbad, CA) supplemented with 10% fetal bovine serum and 100 units/mL penicillin (HyClone, Logan, AR).

### U87GM orthotopic model

The BALB/c nude mice (male, 4–6 weeks old) used in this study were supplied by the Department of Experimental Animals (Daping Hospital, Third Military Medical University, Chongqing, China). All animal use protocols were performed according to the international principles of laboratory animal care and were approved by the Animal Use Subcommittee of Daping Hospital, Third Military Medical University. Anesthetized mice were mounted dorsally with their heads fixed in a stereotaxic apparatus (RWD Life Science, Shenzhen, China). A 5.0 µL cell suspension containing 5 × 10^5^ U87MG cells was injected into the brain using a 20.0 µL microsyringe. The microsyringe was fixed stereotactically to vertically penetrate 4.0 mm into the brain mass, 2.0 mm to the right of the midline suture and 2.0 mm posterior to the outer canthus and was then retracted 0.5 mm. The cell suspension was then injected slowly over a period of 5 min. After injection, the microsyringe was held in place for 8 min and then retracted.

### BEV and TMZ administration

**Figure 8:**
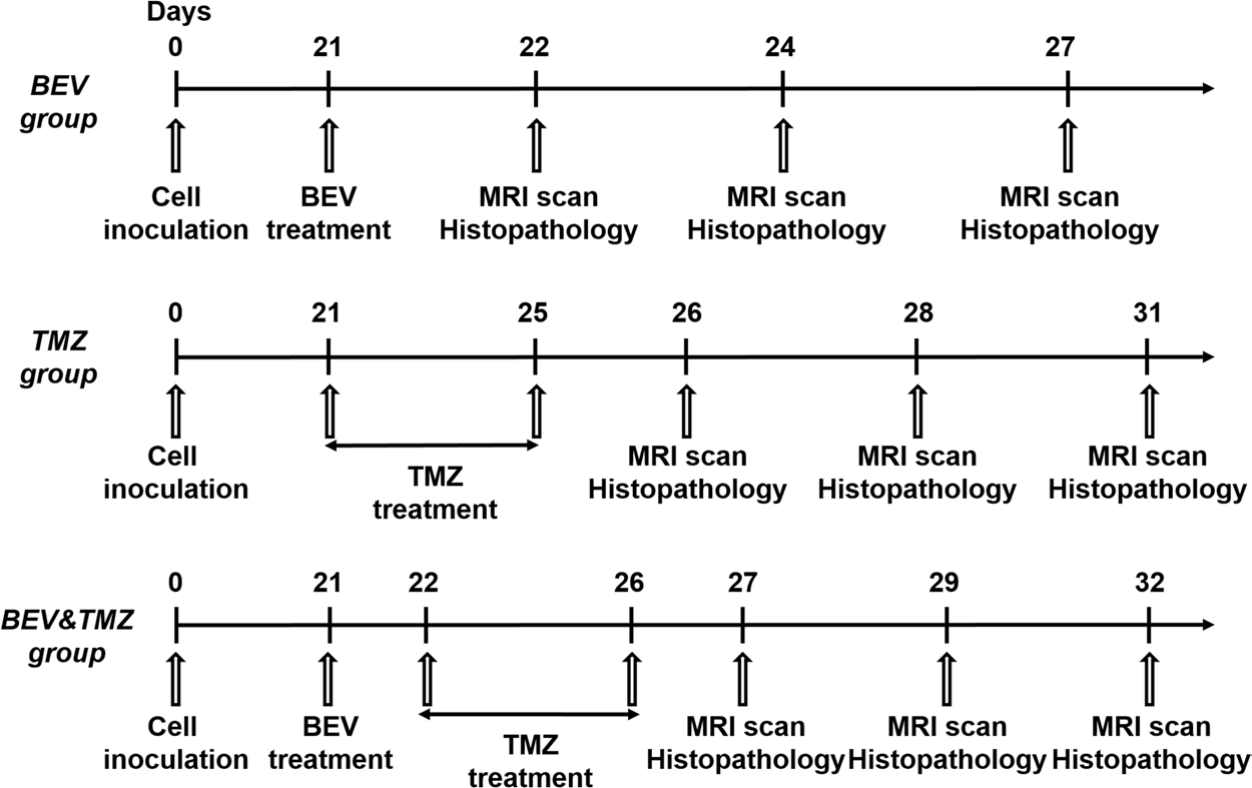
Drug treatment protocols and checkpoints.

Tumor-bearing mice were randomly divided into three groups. Drug administration was performed on day 21 after cell inoculation. MRI scanning were performed prior to treatment (Supplementary Dataset 1). The experimental groups were orally treated with 50 mg/kg TMZ (Merck, NJ, USA) for 5 consecutive days, intravenously administered 15 mg/kg BEV (Avastin, Roche Pharma Ltd, Mannheim, Germany), or administered a combination of TMZ and BEV (TMZ treatment occurred 24 hours after the administration of BEV). The control groups were administered saline in the same way with corresponding experimental groups. The experimental groups (5 mice) and corresponding control groups (6 mice) were examined at 1, 3 and 6 days after treatment. The process is shown in Fig. 8.

### MRI scanning

A Bruker 7.0 T MRI scanner for small animals (Biospin 70/20, Bruker, Ettlingen, Germany) with a head surface coil was used. Animals were fixed on a scanning flat designed for small animals and scanned at 1, 3 and 6 days after treatment. Using the following MRI settings: T2WI, Turbo-RARE sequence, repetition time/echo time = 4000 ms/45 ms, and FOV = 25 × 25 mm, matrix sizes = 256 × 256, and slice thicknesses = 0.5mm. DCE-MRI was performed with the following settings: FLASH sequence, repetition time/echo time = 25.0 ms/1.8 ms, FOV = 25 × 25 mm, matrix sizes = 128 × 128, slice thicknesses = 0.5mm, slices = 5, and flip angle = 5°/15°/20°/30°/15°. Flip angles of 5°, 15°, 20° and 30° were used to perform pre-contrast scans, after which 15° was used to perform dynamic contrast scans. A gadolinium-based contrast agent (OMNISCAN, GE Healthcare, Shanghai, China) was administered via tail-vein injection at a dose of 0.1 mmol/kg manually within 3 s.

### MRI data processing

DCE-MRI data were processed after being imported into Omni Kinetic (GE Healthcare, Shanghai, China). K^trans^ pseudo-color images were calculated following a “reference region” model proposed by Cárdenas-Rodríguez^41^. Then, the K^trans^ of the tumor was calculated using the hot-spot method^42^: Five regions of interest (ROIs) with higher K^trans^ values were chosen in the tumor area, and the K^trans^ value of the tumor was represented by the average K^trans^ value of the five ROIs. K^trans^ values were measured by two experienced colleagues with MRI data analysis experience who were blinded to the group information and pathological results.

### Histopathological analysis

After MRI scanning, tumor-bearing mice were immediately sacrificed using an overdose of anesthesia. Brains were harvested, and a fraction of tumor tissue was randomly selected and divided into pieces of approximately 1 mm^3^ and fixed with 2.5% glutaraldehyde for transmission electron microscope analysis according to the standard procedures described previously^5^. The other tumor tissues were fixed with 4% paraformaldehyde and embedded with paraffin. Paraffin blocks were sectioned to perform H&E and immunohistochemical staining. The antibodies used were raised in rabbits against the following antigens in humans: CD34, glial fibrillary acidic protein (GFAP) and Tenascin-C (Abcam, Cambridge, UK). Two-micron-thick serial sections were used for immunohistochemical staining after dewaxing in xylene. Antigen retrieval was performed in a boiling citric acid solution (pH 6.0) for 2 min, and then slices were washed with PBS after natural cooling. H_2_O_2_ (10%) and goat serum were used to block endogenous peroxidase activity and nonspecific antigens, respectively. Each slice was incubated overnight with solution containing the primary antibody at 4 °C. Specimens were then washed with PBS and incubated with HRP-conjugated goat anti-rabbit secondary antibodies at 37 °C for 30 min. DAB was used to visualize antigens. Dual CD34-periodic acid-Schiff (PAS) staining was performed as described previously^43^ after CD34 immunohistochemical staining. Then, slides were counterstained with Mayer’s hematoxylin and cover slipped with a permanent mounting medium.

### Histopathological quantification

Histopathological quantification was performed by two colleagues who were experienced in histopathological analysis and blinded to the group information. Vascular co-option consisted of GFAP-positive glioma cells circumfusing CD34-positive tubes to form vascular niches^16^. Five fields with maximum co-option were selected under a 100× light microscope. The amount of vascular co-option in the tumor was represented by the average amount of vascular co-option in the five fields.

CD34-positive folds across the lumens of newly formed blood vessels were the key feature of intussusceptive microvascular growth^44^, which was quantified as the average number of folds in 10 lumens with more folds in the tumor area.

Specialized Tenascin-C-positive endothelial cells mediated sprouting angiogenesis^45,46^. Five fields with maximum sprouting angiogenesis were selected using a 200x light microscope, and the number of Tenascin-C-positive stalk cells was counted. Sprouting angiogenesis was quantified as the average number of Tenascin-C-positive stalk cells in the 5 fields.

Vascular mimicry was characterized by lumen-like structures with PAS-positive and CD34-negative basal membrane tissues^47^. We quantified vascular mimicry in the whole tumor region.

Five fields with maximum CD34-positive lumens were selected using a 200X light microscope. The MVD was quantified as the average number of CD34-positive lumens in the 5 fields.

### Statistical analysis

SPSS 19.0 (IBM, Armonk, NY, USA) was used for the statistical analysis, and data are represented as the means ± SD. Image features and pathological results reproducibility were assessed using the intraclass correlation coefficient (ICC). Two sample t-tests were used to compare inter-group differences. Spearman correlation analysis was used to compare the correlation between K^trans^ and neovascularization patterns. A value of P < 0.05 was considered significant.

## Electronic supplementary material

Supplementary Information
