# Supplementary material for: Aberrant glioblastoma neovascularization patterns and their correlation with DCE-MRI-derived parameters following temozolomide and bevacizumab treatment
Source: Sci Rep. 2017 Oct 24;7:13894. doi: 10.1038/s41598-017-14341-9 (PMC5654943; doi:10.1038/s41598-017-14341-9)
Supplement: Supplementary file 1 — Supplementary Information [file 41598_2017_14341_MOESM1_ESM.doc]

**Aberrant glioblastoma** **neovascularization patterns and their correlation with DCE-MRI-derived parameters following temozolomide and bevacizumab treatment**

Wei Xue^1+^, Xuesong Du^1+^, Hao Wu^1^, Heng Liu^1^, Tian Xie^1^, Haipeng Tong^1^, Xiao Chen^1^, Yu Guo^1^, Weiguo Zhang*^1 2^

^1^Department of Radiology, Institute of Surgery Research, Daping Hospital, Third Military Medical University, Chongqing, 400042, China.

^2^Chongqing Clinical Research Center for Imaging and Nuclear Medicine, Chongqing, 400042, China.

*Corresponding author: Weiguo Zhang ([wgzhang01@163.com](mailto:wgzhang01@163.com))

^+^These authors contributed equally to this work.

1. MRI scan results prior to treatment.

|  | Volume (mm^3^) | | | K^trans^ (min^-1^) | | |
| --- | --- | --- | --- | --- | --- | --- |
|  | Original mice (n=24) | Added mice  (n=9) | *P* value | Original mice (n=24) | Added mice  (n=9) | *P* value |
| BEV group | 10.188±1.832 | 9.875±1.683 | 0.659 | 0.323±0.086 | 0.346±0.045 | 0.454 |
| TMZ group | 8.977±1.445 | 8.964±1.597 | 0.982 | 0.212±0.048 | 0.224±0.026 | 0.485 |
| BEV&TMZ  group | 12.487±2.236 | 11.093±1.963 | 0.110 | 0.557±0.102 | 0.531±0.069 | 0.487 |
